# A Holistic Investigation of the Relationship between Digital Addiction and Academic Achievement among Students

**DOI:** 10.3390/ejihpe13100143

**Published:** 2023-09-22

**Authors:** Tijen Tülübaş, Turgut Karakose, Stamatios Papadakis

**Affiliations:** 1 Faculty of Education, Kutahya Dumlupınar University, Kütahya 43100, Türkiye; tijen.tulubas@dpu.edu.tr; 2 Department of Education, University of Crete, 74100 Rethymno, Greece; stpapadakis@uoc.gr

**Keywords:** social media addiction, smartphone addiction, internet addiction, technology addiction, academic achievement, academic performance, science mapping

## Abstract

Digital addiction (DA), an umbrella term referring to addiction to any type of digital media, such as the internet, smartphone, digital games, and social media, is a significant factor influencing students’ academic achievement (AA). Many scholars have contributed to this line of research from around the world. Nevertheless, the literature lacks a holistic investigation of how the DA–AA research field evolved, which could guide future studies. The current study aims to address this void and conducts a combined bibliometric and science mapping analysis of research addressing the relationship between DA and AA. Data were retrieved from the WoS database, considered one of the optimal databases for such studies with its comprehensive coverage of quality journals. One hundred eighteen articles were included in the final dataset and were analyzed using the SciMAT software, which allowed analysis over three consecutive periods and yielded comparable results regarding the conceptual and thematic evolution of the DA–AA domain. The results indicated an increased research interest in the topic, especially during the last five years. The science mapping analysis showed that the most-studied types of addiction were smartphone addiction for the first two periods and social media addiction for the last. Research in the DA–AA domain which focused on understanding the addictive use of smartphones during the first period, evolved to address factors such as self-efficacy or life satisfaction leading to smartphone addiction and lower grades. During the third period, the number of factors addressed gained significant variety and covered family-related and personal factors.

## 1. Introduction

Digital technologies have permeated every aspect of peoples’ lives over the last two decades and fundamentally changed how they communicate, shop, entertain, work, study, and access and use information [1,2] (As the use of computers, the internet, tablets, and smartphones have become ubiquitous and crucial for the functioning of modern society [3,4,5], whether these technologies are beneficial or detrimental have become a matter of concern [6].

The pervasion of digital technologies has yet to escape the attention of educational scholars, considering the potential benefits they might offer for a 21st-century education [7], which inevitably takes place in a fast-changing environment entwined with technology. Considering these technological devices as vital educational tools, technology-integrated classrooms and technology-enhanced instruction were advocated [8]. Indeed, some studies revealed several educational benefits; for instance, the internet was found to support education by enabling more information access, better visual intelligence skills, and enhancement of teacher–student communications [9], social media such as Twitter, Facebook, or Telegram were considered to facilitate learning processes through engaging students in classroom activities, enhancing their capacities to share information, collaborate with peers, arrange study groups, and process information [10,11], as well as helping students to form and maintain social capital, build social networks, and increase social interaction [12,13].

Although digital technologies facilitate many daily operations with the convenience and diverse functions they provide, research showed that their overuse could create serious problems in personal, family, and social well-being [14,15,16], mainly through causing digital addiction (DA). DA is used as an umbrella term to refer to addiction to any digital media such as the internet, computers, smartphones, video games, and social media [17,18]. For instance, a recent study at a global level demonstrated that one fourth of the general population could be experiencing at least one subtype of DA [19], and the already expanding rate of DA is considered to be consolidated by the influence of COVID-19 pandemic [15,20,21].

Research supports that digital media is currently used pervasively by student populations, which makes them vulnerable to DA and its negative consequences [22,23,24,25]. For instance, a review of student social media use showed that problematic internet use had a significant negative effect on academic performance [26]. Similarly, compulsive internet use was shown to distract students’ attention from educational activities and cause a significant loss of time, with adverse effects on academic achievement [27].

DA is now assumed to be a serious threat to the development and education of new generations because students of all ages are now in danger of being overexposed to these technologies as they are born into a world where they interact, play, communicate, and learn utilizing these technologies [28]. Giedd [29] even emphasizes that the ‘desire for digital media is exquisitely aligned with the biology of the teen brain and our evolutionary heritage and adolescents’ hunger for human connectedness, their appetite for adventure, and desire for information turns digital media into a natural allure for them’ (p. 127). As such, students become one of the most vulnerable groups to DA and its negative consequences, such as mental, psychological, physiological, sociological, and educational impairments [4].

In parallel with the exponentially growing literature on addiction to digital technologies [18,30], serious concerns have arisen among educational scholars concerning student DA and its harmful impact on their academic achievement (AA). Numerous studies have made important notifications regarding their association and accumulated a fast-growing knowledge base regarding their relationships. Some studies even addressed the aggregated findings from some of this research and conducted systematic reviews to manifest a more global, evidence-based relationships between a particular type of addiction (e.g., internet addiction) and student AA [6,26,31,32,33,34]. Although these studies made a significant contribution to the field, they all addressed one particular sub-type of digital addiction, and none focused on the development and evolution of this scientific knowledge domain experiencing constant changes due to rapid breakthroughs in digital technologies. However, bibliometric reviews aiming to analyze the intellectual lineage, cognitive structure, and state-of-the-art knowledge in a scientific knowledge domain are currently considered significant for providing a holistic and meaningful understanding of the linkages between the works of several researchers over time, and thus guiding the future development of the field through identifying research gaps or opportunities as well as delineating the thematic patterns underlying its evolution [35,36,37,38].

Considering this gap in the literature regarding the intellectual evolution of research addressing the intersection of student DA and AA, the current study performs a bibliometric performance and science mapping analysis of this scientific domain. While the former analysis aims to conduct a quantitative measure of contributions made by various scientific actors, the latter analysis aims to delineate the thematic structure and growth of the field as well as the evolving research trends over time. With this global purpose, the current study particularly addresses the following research questions (RQ):

RQ1: What is the volume and growth trajectory of scholarship on student DA and AA?
RQ2: What journals, authors, articles, and countries have evidenced the most significant impact in the DA–AA research field?
RQ3: How have the relationships established between DA and AA evolved over time?
RQ4: What are the salient aspects and research frontiers in the DA and AA domain?
RQ5: What are possible future research directions in the DA–AA domain?

Investigating the DA–AA knowledge base within the framework of these research questions, the current study contributes to the literature by manifesting the boundaries and growth trajectory of knowledge in this field [39], providing insights into the growth of thematic interests and networks across periods of time [40,41], and establishing a scientific base for evaluating existing and forthcoming research frontiers as well as identifying the loose ends that warrant future investigation [42].

### Conceptual Background

DA is an overarching term used to describe compulsive, obsessive, and excessive use of digital devices, digital technologies, and digital platforms (e.g., smartphones, computers, the internet, video or online games, and social media) [17,43,44] and refers to an addiction to not only online activities but also offline activities on digital devices [19,45]. DA, with all its types, has become an emerging domain in the behavioral addiction literature, considering that over-exposure to technology can lead to dependence on digital devices/media, yield behavioral symptoms similar to any addictive disorder, and threaten the well-being of the user [6]. It is even considered that DA could be the greatest non-substance addiction facing people in the 21st century [46].

Scholars assert that DA develops through the exact mechanisms and with the same reasons that trigger other types of behavioral addictions (e.g., gambling, drug addiction, and smoking), and ‘what happens in the brain of digital addicts can be assimilated to what happens in any form of addiction’ (p. 5) [47]. Like in other forms of addictions, the intermittent rewards provided by the use of digital devices are considered to activate reward circuits in the human brain and eventually form a dependency on the content and activities offered, such as the games, social network services, or push notifications [48,49].

Digital addicts are also considered to display the already determined six components of behavioral addiction [12,47,50,51]: salience (dominating the user’s thinking and behavior), tolerance (spending more extended periods to reach a satisfactory level), mood modification (changes or improvement in the user’s mood), relapse (the user’s inability to control or stop using), conflict (problems in relationships within work, family, or education), and withdrawal (feeling of discomfort when the activity is reduced or stopped). However, the indispensable nature of digital technologies in contemporary society turns their use into a double-edged sword offering numerous benefits and harms at the same time, which perhaps frames digital addiction as a separate domain of research in the broader addiction literature.

In the existing digital addiction literature, a particular line of research addressed the outcomes of DA for student populations, and investigations into the several types of DA have provided significant correlations between DA and AA, evidencing that DA could impair AA utilizing several factors. One frequently mentioned factor is multitasking, which means that students engaged in digital media are often forced to simultaneously execute two or more tasks, which distracts students’ concentration from their studies [12,52]. For one thing, multitasking might lead to cognitive overload as activities such as chatting, following status updates, generating content, or forming networks require serious cognitive resources [11]. As resource allocation theory explains, cognitive resources are limited, and their unbalanced use results in opportunity costs. Individuals eventually end up paying more vigorous attention to one task than others.

When applied to the educational context, students engaged in such multitasking can lose concentration or interest in learning and deploy weaker effort on academic tasks. Similarly, increased cognitive load is likely to cause serious attention distraction, abstain even the distinguished students from implementing appropriate learning strategies, and lower their AA [51,53,54,55,56].

In addition to multitasking and related attention deficiency problems, the frequent use of digital technologies for long periods also results in other problems such as cognitive fatigue, sleep problems, inability to complete academic responsibilities such as homework, urgency for non-stop entertainment, academic procrastination, and mind-wandering, all of which are detrimental to academic performance and achievement [25,27,57,58].

Given that the digital environments are rapidly becoming more diverse, concepts used for the digital world necessitate constant updates [59], DA is now increasingly preferred as a term to refer to common patterns, symptoms, and outcomes resulting from the problematic or pathological use of digital media [60]. Although early research, particularly emanating from the fields of psychology or psychiatry, frequently preferred terms like problematic or pathological use instead of addiction, the concept of DA has become widespread especially in the last decade [59,61], and have already underlined as a growing problem with several physiological, psychological, and social harm, particularly on student populations [47,62]. Considering that students now devote more time than ever to distractions from the digital world [63], and digital media has become a significant means of adolescents’ identity formulation [64], the academic outcomes of DA has also become a significant issue in the educational literature.

## 2. Materials and Methods

With the purpose of delineating the bibliometric performance and conceptual evolution of scholarship addressing student DA–AA relationship, we conducted a bibliometric performance analysis and science mapping analysis of published research in the field. As previously declared by some scholars [65,66], bibliometric performance analysis is used to yield results with regard to the volume and growth trajectory of the field as well as the journals, authors, articles, and countries that made the most significant contribution to the field. Science mapping analysis, on the other hand, helps delineate the thematic trends, research trajectories, and conceptual evolution of the research field. In the current study, the first analysis was performed in response to RQ1 and RQ2, while the latter was performed in response to RQ3, RQ4, and RQ5.

### 2.1. Data Acquisition

In studies using bibliometric and science mapping analysis, data are frequently searched and extracted using digital databases such as Google Scholar, WoS, or Scopus. The WoS database was preferred in the current study to search and extract data since WoS indexes high-quality publications, particularly in the fields of social sciences, arts, and humanities, and provides appropriate data for bibliometric studies [67]. During this stage, as suggested by Hallinger and Kulophas [39], we first searched data on WoS, evaluated and extracted the raw data according to inclusion/exclusion criteria (see Table 1), and eventually prepared the finally agreed metadata for analysis.

We conducted a search on the WoS database on 25 January 2023, using the following keyword string:

*TS = (“digital addiction” OR “internet addiction” OR “social media addiction” OR “smartphone addiction” OR “mobile phone addiction” OR “digital media addiction” OR “virtual addiction” OR “technology addiction” OR “computer game addiction” OR “gaming addiction” OR “mobile addiction” OR “digital game” OR “virtual game” OR “online game” OR “social network” OR “online shopping addiction” OR “cybersex addiction” OR “online movies” OR “social media” OR “gadget addiction” OR “mobile apps addiction” OR “internet gaming” OR “gaming disorder” OR “sms addiction” OR “mobile media” OR “problematic use” OR “problematic smartphone use” OR “problematic social media use” OR “game addiction” OR “digital leisure” OR “selfie addiction”) AND TS=(“academic achievement” OR “academic performance” OR “academic success” OR “academic attainment”)*

The keywords in the search string were selected after a comprehensive literature review on DA–AA-focused research. As mentioned earlier, research addressing the behavioral symptoms or outcomes of the disabling/harmful use of digital technologies included a large variety of concepts. While preparing the search string, we paid attention to this diversity and aimed to include a broad scope of keywords so that we could access a larger dataset addressing the intersection of DA–AA. We also consulted two other field experts (one in addictions and the other in education) before finalizing the search string and received the approval of both.

**Figure 1:**
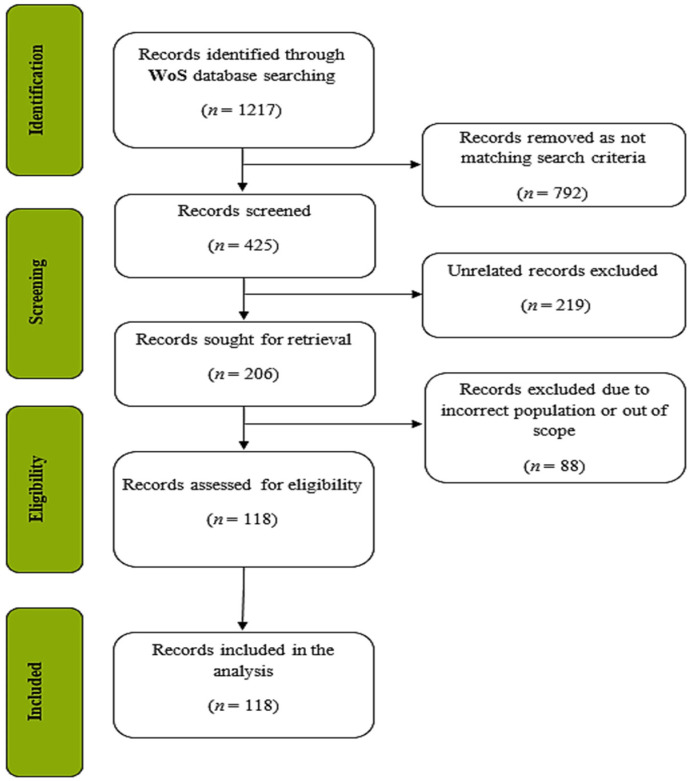
PRISMA flow diagram.

We illustrated the process of data acquisition using Moher et al.’s [68] Preferred Reporting Items for Systematic Reviews and Meta-reviews (PRISMA) diagram presented in Figure 1.

The search first yielded 1217 publications in total. We excluded 792 publications since they were not compatible with our inclusion criteria (Table 1). At this stage, 425 publications remained for analysis. Then, we scanned through the titles of these 425 publications and decided to exclude 219 publications from the dataset since their titles were found not to be directly related to either DA or AA or their relationship. Next, we skimmed through the abstracts of the remaining 206 publications and selected 118 articles for our analysis.

### 2.2. Data Analysis

First, we transferred the bibliographic data such as title, authors(s), abstract, keywords, citations, year, journal, and country belonging to the 118 articles selected for analysis into the SciMAT program and prepared them for analysis. For instance, we manually combined key terms with similar meanings, such as ‘addiction and addictions’ and ‘self-control and self-control’ so as to improve the quality of the subsequent science mapping analysis [37,69]. Then, we performed an overall bibliometric performance analysis so as to determine the distribution of publications by year, the accumulated number of publications, and the average citations received by each publication [41]. Next, we performed a science mapping analysis to determine the thematic structure and evolution of the DA–AA research field.

We conducted the analysis using the SciMAT software tool, version 1.1.04 [69]. SciMAT allows for defining and visualizing thematic trends in a research field, exhibiting its thematic evolution across different periods of its development, and thus determining the scope and performance of scholarship in the field. SciMAT analysis is compelling since it enables observing the evolution of a research field over sequential periods [35,67,69,70,71] and helps reveal the structural and dynamic aspects of the field in a longitudinal manner.

**Figure 2:**
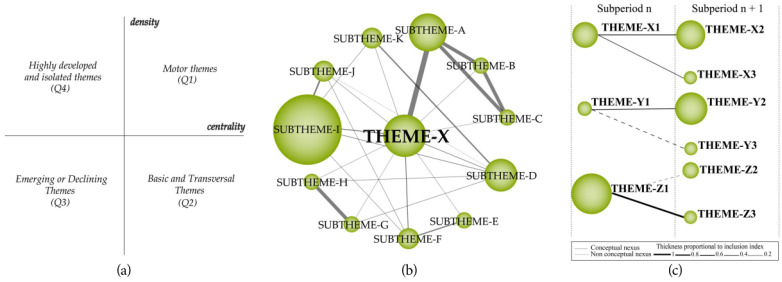
(**a**) Strategic diagram, (**b**) Thematic Network Structure, (**c**) Thematic Evolution Structure [74].

During the science mapping analysis, the conceptual link between themes from different periods was calculated using the inclusion index [(the equation: Ii = #(U∩V)/min(#U, #V)], as suggested by Börner et al. [72] and Sternitzke and Bergmann [73]. This conceptual link was created by connecting the U and V themes via combining common keywords (i.e., conceptual linking), and the results are presented in a thematic evolution map. As the number of common keywords between the clusters across periods increases, the thematic evolution becomes more evident. The science mapping analysis was performed through the following stages [65,67,69]:

(i) *Identification of research topics*: For each period of analysis, we first created a standardized network of common words using keywords in the dataset. We then applied a clustering algorithm created over the co-occurrence of the keywords to a normalized network of common words. This process allows for identifying the research trends and themes in each period.
(ii) *Visualization of research themes and thematic networks:* The themes identified during the previous stage are presented in the strategic diagram and the Thematic Network Structure. The strategic diagram is two-dimensional (the x-axis represents the centrality values while the y-axis represents the density values), and includes four quadrants. The centrality value is formulated as c = 10 × Σekh and shows the strength of the interaction between two clusters. The density value is formulated as d = 100 (Σeij/w) and shows the strength of the interaction between keywords within a theme. As a result, research themes yielded from the analysis are presented in one of these four quadrants based on co-word and h-index analyses (see Figure 2a for an example). The following labels are used to define these themes in each quadrant:
  (a) *Motor themes (Q1):* Their values of centrality and density are both high, which shows that these themes are highly developed and have a significant role in the development and structuring of scholarship in the field;
  (b) *Basic and transversal themes (Q2):* Their values of centrality are high while the values of density are low, which shows that these themes are relevant for research in the field but they are not sufficiently developed. These themes bear the potential to become motor themes in subsequent periods;
  (c) *Emerging or declining themes (Q3):* These themes have low centrality and density values, which shows that they are insufficiently developed and mostly marginal to the research field under analysis;
  (d) *Highly developed and isolated themes (Q4):* Their values of centrality are low while the values of density are high, which shows that they are highly specialized on their own and remain peripheral to the field although they are sufficiently developed. This might result from their lack of the appropriate theoretical background for that particular field of research.

Figure 2b shows an example of a *thematic network structure* that illustrates the emergence and evolution of strategic themes as well as related sub-themes for each of these strategic themes. The central theme in the structure is labeled using the most significant keyword, and the related keywords are presented around this central theme. The volume of the spheres reflects the number of publications and the thickness of the lines reflects the strength of their interrelationship.

Figure 2c shows an example of a *thematic evolution map* that illustrates a set of themes that emerged across different periods as well as the interrelationships between these themes. Thus, the map visualizes the evolution of themes throughout the development of the particular research field. When the themes from each period share the same keywords, this is shown with solid lines. When the theme names share some common keywords, this is shown with dashed lines. As the number of publications representing each theme increases, the circles become larger. As the strength of the relationship between themes increases, the lines become thicker.

Another significant stage in bibliometric science mapping analysis is the period creation, which saves the data from uniformity [37], and allows for the longitudinal, comparative analysis of the conceptual/thematic evolution of the research field [65]. While determining the periods of analysis, Cobo et al. [65] suggest using two approaches: (1) focusing on key changes in the field and (2) ensuring the inclusion of a sufficient and balanced number of documents in each period. They also underline that including an almost balanced number of documents is compulsory to obtain rigorous results. In the current study, the periods were formed according to the number of publications as well as the empirical development of the field, such that Period 1 (2009–2016) comprised 22 articles, Period 2 (2017–2019) comprised 38 articles, whilst Period 3 (2020–2022) comprised 47 articles.

## 3. Results

### 3.1. Overall Bibliometric Analysis

First, we performed a bibliometric performance analysis to illustrate the global impact of publications [41]. The analysis yielded the yearly distribution of articles, the accumulated number of publications and citations received per article, the most cited authors/articles, as well as the most productive countries.

#### 3.1.1. Publication Trends

**Figure 3:**
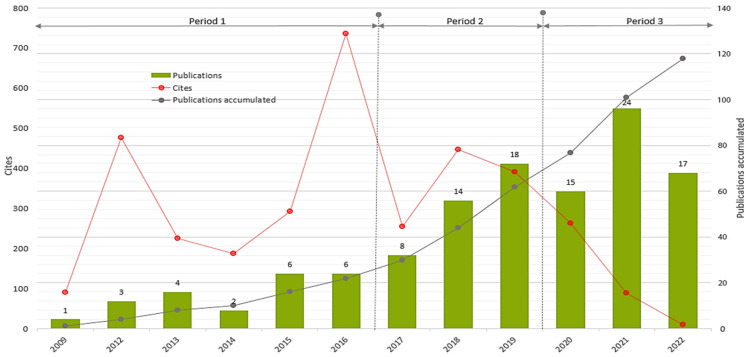
Distribution of publications and citations over time (2009–2022).

The yearly distribution of articles, their accumulated numbers, as well as the average number of citations received per article are shown in the graphical representation in Figure 3.

As shown in Figure 3, the first study investigating the relationship between DA and AA was published in 2009, and publications began to increase gradually in 2012. Research in this field has accumulated a relatively more robust knowledge base since 2018. On the other hand, articles published in 2016, 2012, and 2018, respectively, have received the highest citation rates, which implies their more significant contribution to the development of the field.

#### 3.1.2. Most Influential Authors

Within the scope of the 118 articles analyzed, the total number of publishing authors was 356, with some authors also having been involved in more than one article. The top 10 most productive authors were listed in Table 2 according to the highest citation rates.

Table 2 shows that Hawi and Samaha have been the leading authors with the highest number of publications and citations (n = 674), followed by Junco with 379 citations received for his single publication. It is noteworthy that many authors contributed to the field with only one article but made a significant contribution by being cited by hundreds of other authors.

#### 3.1.3. Most Influential Journals

The 10 journals that have published the highest number of articles on the DA and AA relationship are listed in Table 3.

Table 3 illustrates that *Computers in Human Behavior* and *Computers & Education* contributed the most to the research field with 13 and 11 publications, respectively. It is noteworthy that journals from various research fields were interested in publishing studies on the DA–AA relationship.

#### 3.1.4. Most Cited Articles

The top 10 articles that received the highest citations among the 118 articles analyzed were listed in Table 4, based on the total citations received.

Table 4 illustrates that the top two articles by Samaha and Hawi [75] and Junco [52] were published in Computers in Human Behaviour journal and received the highest citation rate, corresponding with the results shown in Figure 2.

#### 3.1.5. Most Productive Countries

The top 10 countries with the most publications in the field are listed in Table 5 according to the number of publications.

As shown in Table 5, studies from various countries across the globe were published, although the leading countries were found to be China and the USA. The list indicates a relatively balanced contribution to the field from both Western and Eastern contexts.

### 3.2. Science Mapping Analysis

This section reports the results of science mapping analysis performed using SciMAT: (i) period-based thematic analysis, (ii) overlapping items analysis, and (iii) the thematic evolution analysis.

#### 3.2.1. Scientific Evolution Structure

##### Period 1 (2009–2016)

**Figure 4:**
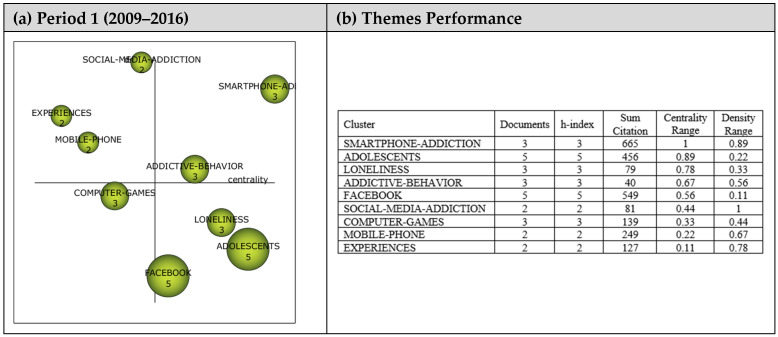
**Period 1** (**a**) Strategic diagram, (**b**) performance analysis.

The analysis for Period 1 included 22 articles, and their analysis yielded nine themes as shown in the strategic diagram in Figure 4. The performance values (i.e., h-index, centrality, and density values as well as the number of documents and sum citations for each theme) are also presented.

A total of nine main themes emerged during the first period (2009–2016). *Smartphone-Addiction* and *Addictive-Behavior* themes emerged as motor themes, contributing to the field’s development. *Social-Media-Addiction*, *Mobile-Phone,* and *Experiences* themes were the highly advanced and isolated themes. The themes in this region are strongly related but do not have the appropriate background or significance for the field. The *Computer-Games* theme was one of the emerging/declining themes, which was likely to have weakened during the relevant period. *Adolescents*, *Loneliness,* and *Facebook* themes were included in the basic and transversal themes, which needed to be developed more despite being related to the field. Nevertheless, five documents each represented *Facebook* and *Adolescents* themes of high significance.

**Figure 5:**
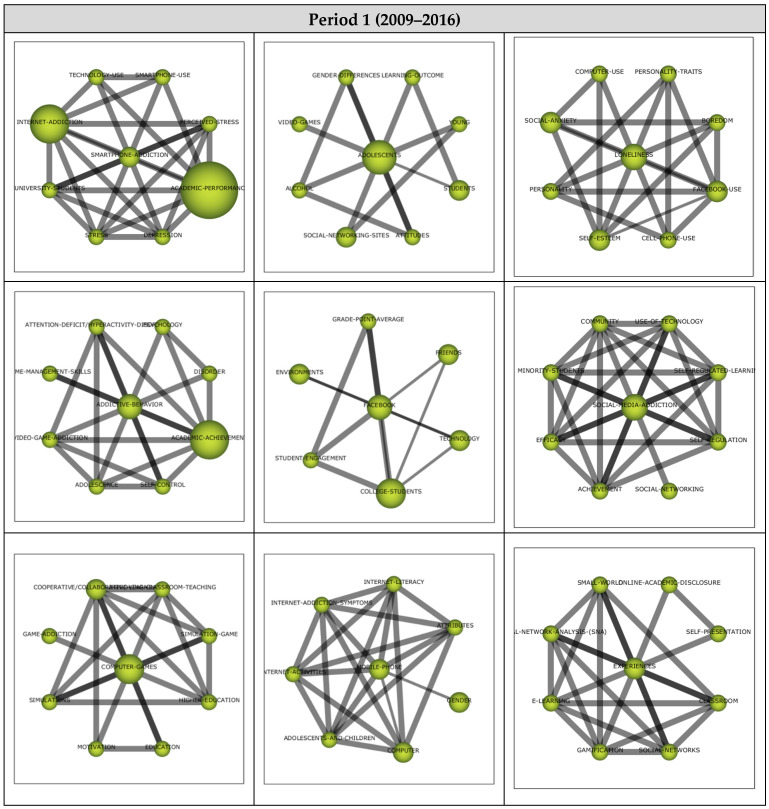
Thematic network structures for Period 1.

Cluster networks (Figure 5) were examined to determine the sub-themes related to the motor themes that emerged during the first period. Accordingly, the central theme of *Smartphone-Addiction* (1, 0.89) was strongly related to *Smartphone-Use*, *Perceived-Stress*, *Academic-Performance, Depression*, *Stress*, *University-Students*, *Internet-Addiction,* and *Technology-Use*, with strong interrelationships among all sub-themes. Studies on Smartphone-Use [51], Perceived-Stress [75], Academic-Performance [33], Depression [76], Stress [75], University-Students [77], Internet-Addiction [78], and Technology-Use [79] illustrate the sub-themes in the Smartphone-Addiction cluster network.

The central theme of Addictive-Behavior (0.67, 0.56) was found to have strong relations with *Psychology, Disorder, Academic-Achievement, Self-Control, Adolescent, Video-Game-Addiction, Time-Management-Skills*, and *Attention Deficit-Hyperactivity-Disorder*. These studies address Psychology [80], Disorder [81], Academic-Achievement [82], Self-Control [83], Adolescent [78], Video-Game-Addiction [76], Time-Management-Skills [84], and Attention Deficit-Hyperactivity-Disorder [83] illustrate the sub-themes of Addictive-Behavior cluster network.

##### Period 2 (2017–2019)

**Figure 6:**
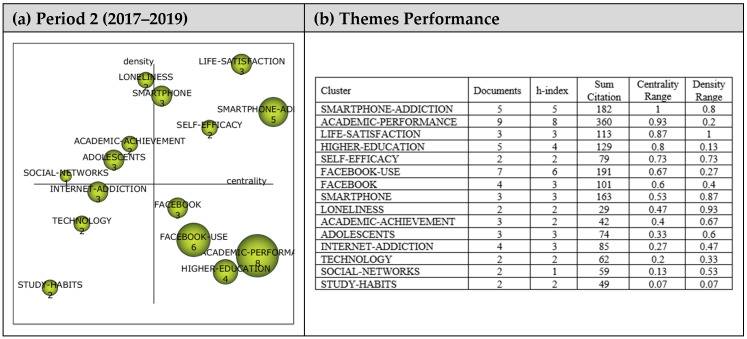
**Period 2** (**a**) Strategic diagram, (**b**) performance analysis.

A total of 15 themes emerged from analyzing the 38 articles in the second period as shown in the strategic diagram in Figure 6. The performance values (i.e., h-index, centrality, and density values as well as the number of documents and sum citations for each theme) are also presented.

A total of four main themes emerged during the second period (2017–2019). *Smartphone-Addiction*, *Life-Satisfaction*, *Self-Efficacy,* and *Smartphone* themes emerged as motor themes that guided research during this period. *Loneliness*, *Academic-Achievement*, *Adolescents,* and *Social-Networks* themes were highly developed and isolated themes. The themes in this region are strongly related but do not have the appropriate background or significance for the field. *Internet-Addiction*, *Technology*, and *Study-Habits* themes were the emerging/declining themes, whilst *Academic-Performance*, *Higher-Education*, *Facebook Use*, *and Facebook* themes were the primary and transversal themes.

**Figure 7:**
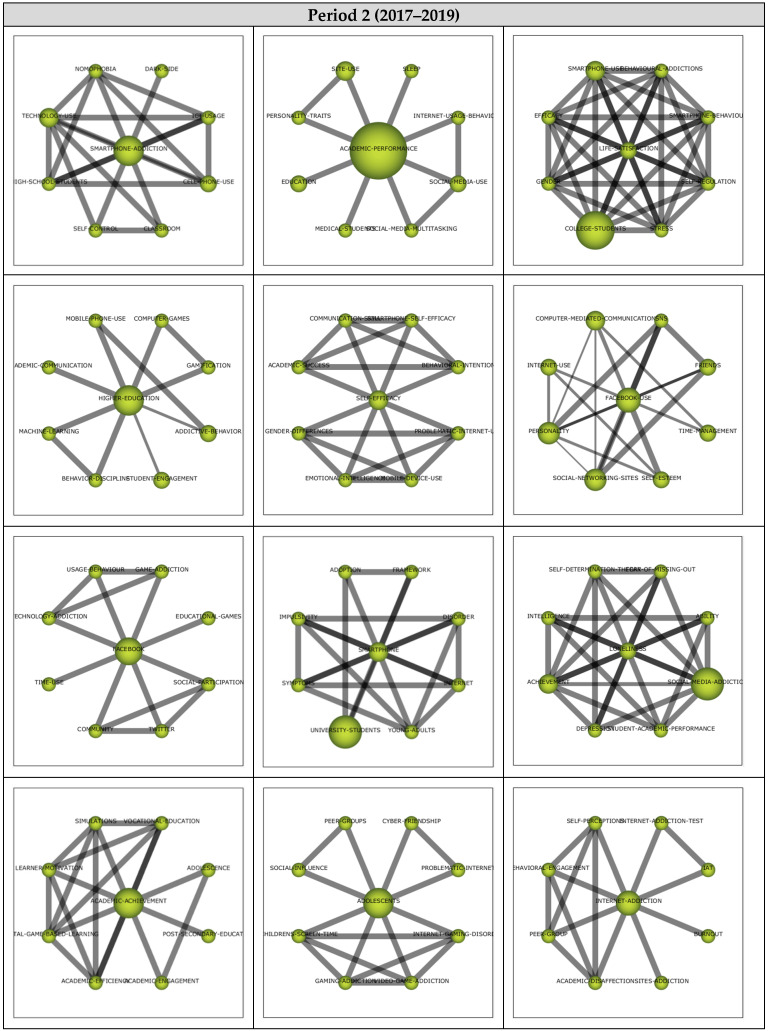

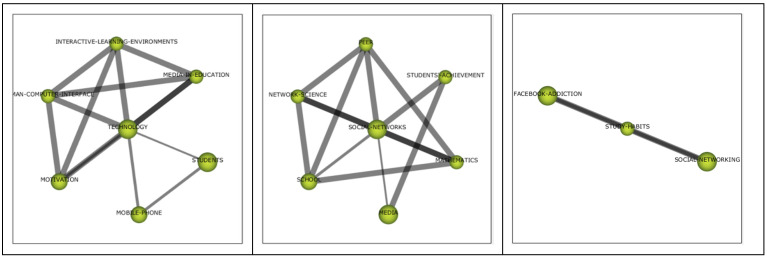
Thematic network structures for Period 2.

Cluster networks of motor themes that emerged during the second period (Figure 7) were analyzed to determine the relevant sub-themes. *Smartphone-Addiction* (1, 0.8) was found to be related to *Dark-Side*, *ICT-Usage*, *Cell-Phone-Use*, *Classroom*, *Self-Control*, *High-School-Students*, *Technology-Use*, and *Nomophobia*. Studies addressing Dark-Side [50], ICT-Usage [79], Cell-Phone-Use [85], Classroom [86], Self-Control [87], High-School-Students [79], Technology-Use [85], and Nomophobia [79] illustrate the sub-themes in the Smartphone-Addiction cluster network.

The Life-Satisfaction theme (0.87, 1) was determined to have a strong relationship with Behavioural-Addictions, Smartphone-Behaviour, Self-Regulation, Stress, College-Students, Gender, Efficacy, and Smartphone-Use. These studies on Behavioural-Addictions [88], Smartphone-Behaviour [88], Self-Regulation [89], Stress [88], College-Students [90], Gender [79], Efficacy [91], and Smartphone-Use [92] illustrate the sub-themes in the Life-Satisfaction cluster network.

The Self-Efficacy theme (0.73, 0.73) was strongly associated with Smartphone-Self-Efficacy, Behavioral-Intention, Problematic-Internet-Use, Mobile-Device-Use, Emotional-Intelligence, Gender-Differences, Academic-Success, and Communication-Skill. These studies addressed Smartphone-Self-Efficacy [90] Behavioral-Intention [90], Problematic-Internet-Use [79], Mobile-Device-Use [77], Emotional Intelligence [79], Gender-Differences [77], Academic-Success [93], and Communication-Skill [90] illustrate the sub-themes in the Self-Efficacy cluster network.

The *Smartphone* theme (0.53, 0.87) was found to have a relationship with *Framework*, *Disorder*, *Internet*, *Young-Adults*, *University-Students*, *Symptoms*, *Impulsivity*, and *Adoption* sub-themes. These studies on Framework [90], Disorder [81], Internet [87], Young-Adults [11], University-Students [58], Symptoms [92], Impulsivity [92], and Adoption [90] are some of the examples of the subthemes in the *Smartphone* cluster network.

##### Period 3 (2020–2022)

**Figure 8:**
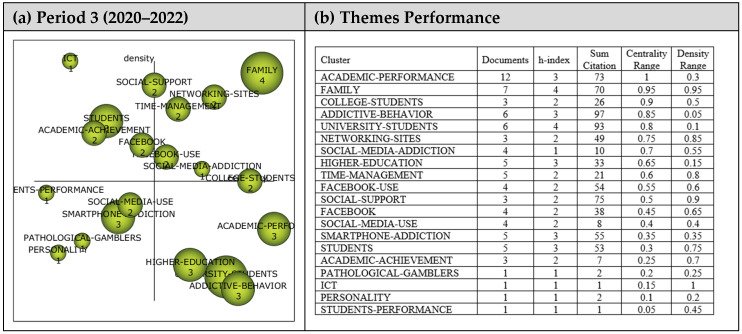
**Period 3** (**a**) Strategic diagram, (**b**) performance analysis.

The analysis for Period 3 included 47 articles and their analysis yielded 20 themes as shown in the strategic diagram in Figure 8. The performance values (i.e., h-index, centrality, and density values as well as the number of documents and sum citations for each theme) are also presented.

The *Academic-Performance* theme emerged as the most significant theme represented by 12 documents. While *Family*, *College-Students*, *Networking-Sites*, *Social-Media-Addiction*, *Time-Management*, *Facebook-Use,* and *Social-Support* emerged as motor themes, *Facebook*, *Students*, *Academic-Achievement,* and *ICT* themes were included in the highly developed and isolated themes. *Social-Media-Use*, *Students-Performance*, *Smartphone-Addiction*, *Pathological-Gamblers*, and *Personality* themes were emerging/declining themes, whilst *Academic-Performance*, *Addictive-Behavior*, *University-Students*, and *Higher-Education* themes emerged as basic and transversal themes.

**Figure 9:**
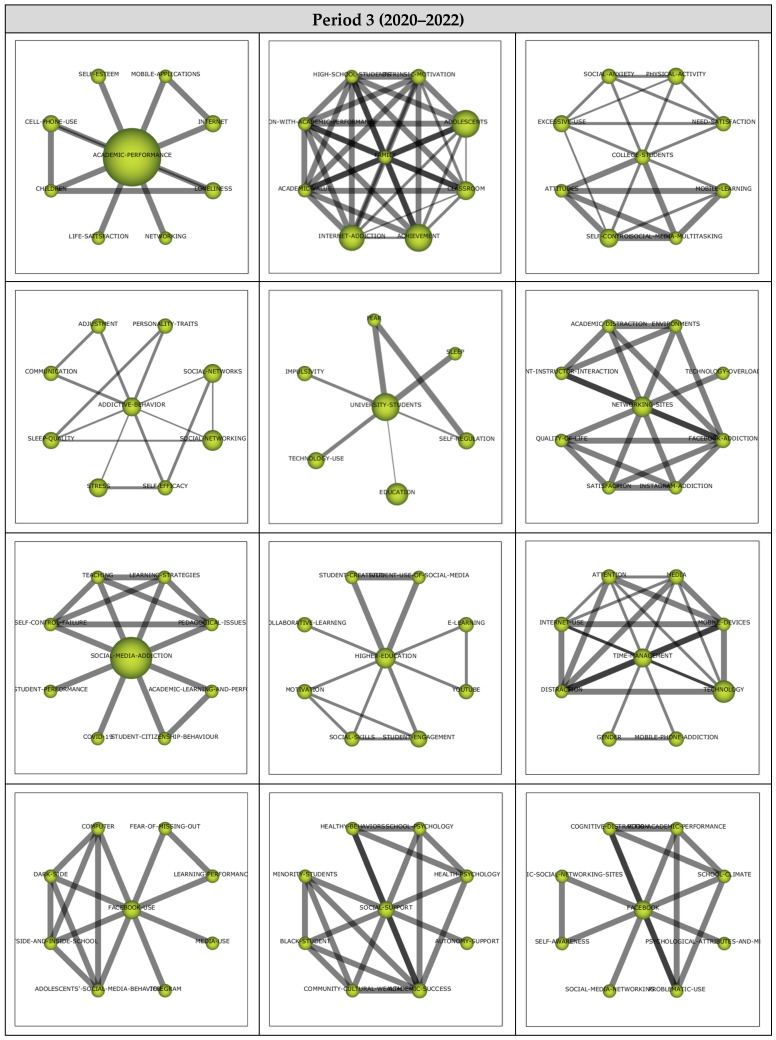

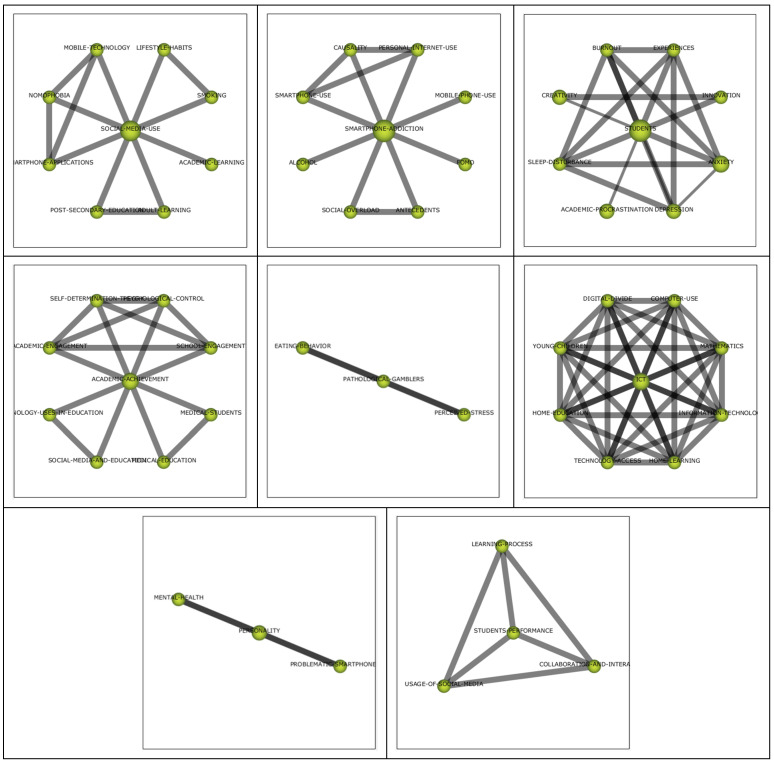
Thematic network structures for Period 3.

Motor theme cluster networks (see Figure 9) were examined to determine the relevant subthemes. The *Family* theme (0.95, 0.95) was found to have a strong relationship with *Intrinsic-Motivation*, *Adolescents*, *Classroom*, *Achievement*, *Internet-Addiction*, *Academic-Value*, *Satisfaction-with-Academic-Performance*, and *High-School-Students*. Studies addressing Intrinsic-Motivation [94], Adolescents [95], Classroom [96], Achievement [97], Internet-Addiction [98], Academic-Value [98], Satisfaction-with-Academic-Performance [98], and High-School-Students [98] illustrate the sub-themes in the *Family* cluster network.

The *College-Students* theme (0.9, 0.5) was determined to be related to *Physical-Activity*, *Need-Satisfaction*, *Mobile-Learning*, *Social-Media-Multitasking*, *Self-Control*, *Attitudes*, *Excessive-Use*, and *Social-Anxiety*. These studies focus on Physical-Activity [99], Need-Satisfaction [97], Mobile-Learning [48], Social-Media-Multitasking [100], Self-Control [101], Attitudes [102], Excessive-Use [103], and Social-Anxiety [104] illustrate these sub-themes in the *College-Students* cluster network.

The *Networking-Sites* theme (0.75, 0.85) was found to be related to *Environments*, *Technology-Overload*, *Facebook-Addiction*, *Instagram-Addiction*, *Satisfaction*, *Quality-Of-Life*, *Student-Instructor-Interaction*, and *Academic-Distraction*. These studies address Environments [105], Technology-Overload [106] Facebook-Addiction [107], Instagram-Addiction [22], Satisfaction [98], Quality-of-Life [22], Student–Instructor-Interaction [107], and Academic-Distraction [107] illustrate the sub-themes in the Networking-Sites cluster network.

The *Social-Media-Addiction* theme (0.7, 0.55) was determined to have relationships with *Learning-Strategies*, *Pedagogical-Issues*, *Academic-Learning-And-Performance*, *Student-Citizenship-Behaviour*, *COVID-19*, *Student-Performance*, *Self-Control-Failure*, and *Teaching*. These studies address Learning-Strategies [100], Pedagogical-Issues [100], Academic-Learning-and-Performance [108], Student-Citizenship-Behaviour [108], COVID-19 [109], Student-Performance [110], Self-Control-Failure [100], and Teaching [100] illustrate the sub-themes in the *Social-Media-Addiction* cluster network.

The *Time-Management* theme (0.6, 0.8) was found to have relationships with *Media, Mobile-Devices, Technology, Mobile-Phone-Addiction, Gender, Distraction, Internet-Use,* and *Attention*. These studies address Media [103], Mobile-Devices [111], Technology [112], Mobile-Phone-Addiction [113], Gender [114], Distraction [111], Internet-Use [97], and Attention [54] illustrate the sub-themes in the *Time-Management* cluster network.

The *Facebook-Use* theme (0.55, 0.6) was determined to be related to *Fear-of-Missing-Out*, *Learning-Performance*, *Media-Use, Telegram*, *Adolescents’-Social-Media-Behavior*, *Outside-and-Inside-School*, and *Dark-Side*. These studies address Fear-of-Missing-Out [115], Learning-Performance [106], Media-Use [107], Telegram [112], Adolescents’-Social-Media-Behavior [95], Outside-and-Inside-School [95], and Dark-Side [95] illustrate the sub-themes in the *Facebook-Use* cluster network.

The *Social-Support* theme (0.5, 0.9) was found to be related to *School-Psychology*, *Health-Psychology*, *Autonomy-Support*, *Academic-success*, *Community-Cultural-Wealth*, *Black-Student*, *Minority-Students*, and *Health-Behaviors*. These studies addressing School-Psychology [116], Health-Psychology [116], Autonomy-Support [97], Academic-Success [34], Community-Cultural-Wealth [34], Black-Student [34], Minority-Students [34], and Health-Behaviors [116] illustrate the sub-themes in the Icluster network.

#### 3.2.2. Overlapping Map

**Figure 10:**
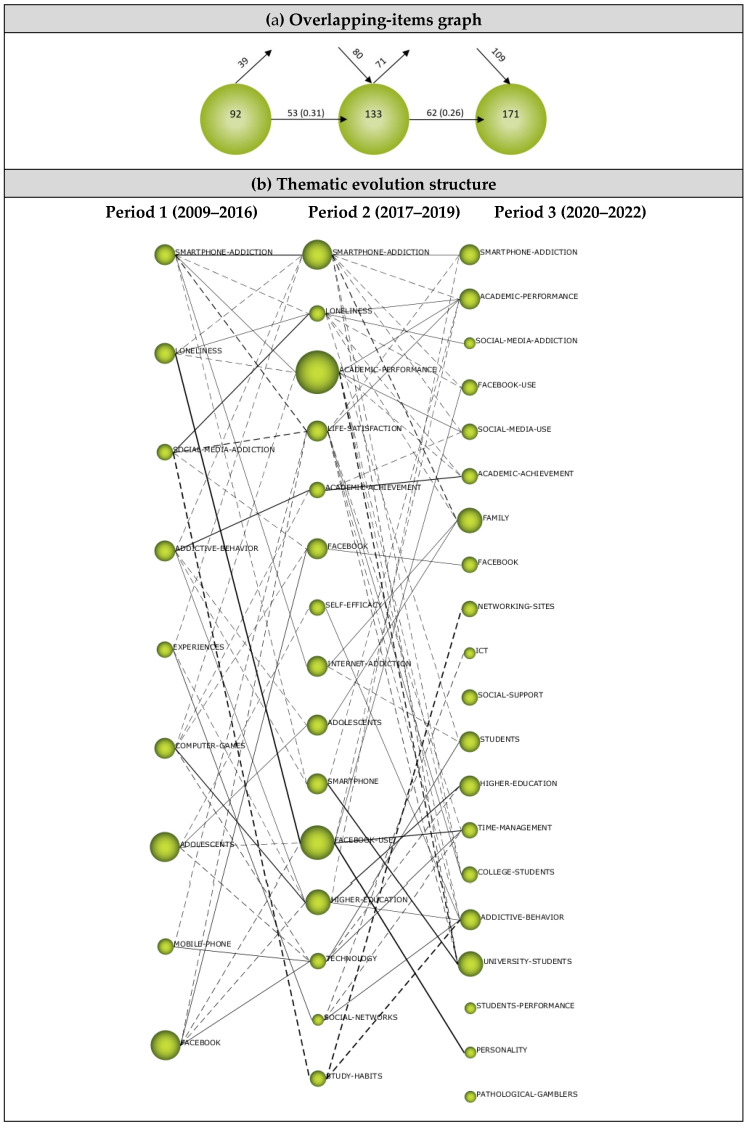
(**a**) Overlapping map, (**b**) Thematic evolution map.

The *overlapping-items graph* shows the number of keywords in each period and highlights keywords that newly appeared, disappeared, or were reused in the next period [117]. The overlapping map in Figure 10a shows that 92 keywords emerged during the first period, 39 were not transferred to the next period, whilst 53 were used. However, 133 keywords appeared during the second period, 62 of which were also used in the third period, whilst 71 were not used. During the third period, a total of 171 keywords emerged. While the number of newly-used keywords was 80 during the second period, it was determined to be 109 during the third period. However, the similarity index was found to have decreased between the sub-periods (from 0.31 to 0.26), which indicates that the number of keywords that newly appeared or lost between periods was high.

The overlapping-items graph revealed that the terminology related to DA–AA research got more robust each year, and new terms continued to emerge across periods. Keywords from left to right, from the first period to the last period, increased from 92 to 171. This significant increase in the number of keywords indicates that themes regarding DA and AA relationships have been diversifying and increasing cumulatively. The increase in the number of keywords added in each period shows that the studies addressing DA and AA are constantly developing and being updated.

#### 3.2.3. Thematic Evolution Structure

The thematic evolution map in Figure 10b illustrates the relationship between the development patterns in the knowledge domains of research addressing the DA and AA relationship over the periods of analysis. The size of the spheres on the map corresponds with the number of articles, and the thickness of the lines connecting the spheres shows the correlation between the themes in the periods [69,118].

The thematic evolution map shows that nine themes emerged during the first period (2009–2016), constituting 18.64% of the articles (n = 22). While four of these themes continued in other periods, five were connected with different themes. The lines connecting the themes between the periods show that these themes shared keywords. While the *Smartphone-Addiction* and *Facebook* themes emerged during the first period and continued their existence for three periods, the *Loneliness* and *Adolescents* themes continued their existence only during the first two periods. The *Smartphone-Addiction* theme was associated with the *Academic-Performance*, *Facebook-Use*, *Social-Media-Use*, *Academic-Achievement*, *Family*, *College-Students*, and *Addictive-Behavior* themes that emerged during the last period. The *Loneliness* theme was associated with *Academic-Performance*, *Social-Media-Addiction*, *Facebook-Use*, *Academic-Achievement*, *Family*, and *Students* themes that emerged during the last period. The *Adolescents* theme was associated with the *Family* theme, whilst the *Social-Media-Addiction* theme had associations with the *Loneliness*, *Life-Satisfaction*, *Facebook*, and *Study-Habits* themes that emerged during the second period. The *Addictive-Behavior* theme was associated with the *Academic-Achievement*, *Adolescent*, *Smartphone*, and *Higher-Education* themes that emerged during the second period, whilst the *Experiences* theme had associations with the *Smartphone-Addiction*, *Higher-Education,* and *Social-Networks* themes from the second period. The *Computer-Games* theme was associated with the *Academic-Performance*, *Academic-Achievement*, *Facebook*, *Higher-Education,* and *Technology* themes, whilst the *Mobile-Phone* theme was associated with the *Life-Satisfaction* and *Technology* themes. The *Adolescent* and *Facebook* themes were observed to have the highest H-index during the first period.

Fifteen themes emerged during the second period (2017–2019), constituting 32.20% of the articles (n = 38). Four of these themes were transferred from the first period, and 11 appeared for the first time during this period, yet all these themes were found to have associations with the themes that emerged during the first period. The *Smartphone-Addiction*, *Academic-Performance*, *Academic-Achievement*, *Facebook*, *Facebook-Use*, and *Higher-Education* themes continued to exist during the third period. The *Smartphone-Addiction* theme was associated with the *Academic-Performance*, *Facebook-Use*, *Social-Media-Use*, *Family*, *College-Students,* and *University-Students* themes that emerged during the last period. The *Loneliness* theme was associated with the *Academic-Performance*, *Facebook-Use*, *Academic-Achievement*, *Family,* and *Students* themes that emerged during the last period. The *Academic-Performance* theme had associations with the *Social-Media-Use*, *Academic-Achievement*, and *University-Students* themes from the last period, whilst the *Life-Satisfaction* theme had associations with the *Smartphone-Addiction*, *Academic-Performance*, *Time-Management*, *College-Students*, *Addictive-Behavior*, and *University-Students*. The *Self-Efficacy* theme was associated with the *Addictive-Behavior* theme, whilst the *Internet-Addiction* theme was associated with the *Family* and *Students* themes from the last period.

The *Adolescent* theme had associations with the *Family* theme that emerged during the last period, and the *Smartphone* theme had associations with *Academic-Performance* and *University-Students* themes. The *Facebook-Use* theme had associations with the *Academic-Performance* and *Personality* themes that emerged during the last period. Similarly, the *Higher-Education* theme was associated with the *Smartphone-Addiction* and *Addictive-Behavior* themes. The *Technology* theme was associated with the *Students, Higher-Education, Time-Management*, and *Addictive-Behavior* themes that emerged during the last period, whilst the *Social-Networks* theme was associated with the ICT, *Time-Management,* and *Addictive-Behavior* themes. The *Study-Habits* theme had associations with the *Networking-Sites* and *Addictive-Behavior* themes from the last period. During the second period, the highest H-index belonged to the *Academic-Performance* theme.

Twenty themes emerged during the third period (2020–2022), comprising 49.16% of the articles (n = 58). The *Smartphone-Addiction*, *Academic-Performance*, *Facebook-Use*, *Academic-Achievement*, *Facebook*, and *Higher-Education* themes were transferred from the previous period, while other themes emerged for the first time (i.e., *Social-Media-Addiction*, *Social-Media-Use*, *Family*, *Networking-Sites*, *ICT, Social-Support*, *Students*, *Time-Management*, *College-Students*, *Addictive-Behavior*, *University-Students*, *Students-Performance*, *Personality*, and *Pathological-Gamblers* themes. All the themes that emerged during the last period were associated with the themes of the second period, except the *Social-Support*, *Students-Performance,* and *Pathological-Gamblers*. The highest H-index during the last period belonged to the *Family* and *University-Students* themes.

## 4. Discussion and Conclusions

The current study conducted a holistic review of the published research addressing the relationship between DA and AA. It delineates the conceptual and scientific evolution of this research field by exhibiting the strategic themes that emerged during the different stages of its evolution and identifies the scientific actors that contributed greatly to the development of this line of research. Our integrated bibliometric and science mapping analysis revealed significant implications, particularly for the future investigation of the DA–AA relationship through highlighting well or under-researched aspects.

The bibliometric analysis was conducted to answer the first two research questions identified earlier (RQ1 and RQ2). The results showed that the number of studies investigating the relationship of DA with AA increased significantly since the first study was conducted in 2009. As a recent but significant phenomenon, the problematic use of digital technologies has raised concerns about its possible adverse effects on students’ learning and achievement. Studies were conducted in various contexts, both in Eastern and Western countries. The science mapping analysis, on the other hand, showed that the themes of investigation evolved in parallel to technological developments and evidence provided.

The science mapping analysis was conducted to address three remaining research questions (RQ3, RQ4, and RQ5), which particularly focused on the structural and dynamic aspects of the evolution of the research field over time. The results revealed that studies on the relationship between DA and AA were primarily focused on smartphone addiction, while research interest in computer game addiction was weakened. Following the rising popularity of smartphones since the beginning of the 2000s thanks to their ever-increasing capacity to offer a multitude of utilities and applications (apps) to address everyday needs such as communication, entertainment, or networking [119], smartphones quickly became an indispensable part of many people’s lives, including children, adolescents, and young adults [120]. Despite numerous advantages, including the offer for not only daily or business life, but also education, such as language learning [121,122] or classroom engagement [123], earlier research raised concerns that the problematic use of smartphones could result in behavioral addiction and could deteriorate the psychology and academic performance of students [51,85,124]. Indeed, when the sub-themes of smartphone addiction were scrutinized, it was evident that this research trajectory mainly addressed university students’ psychological states, such as depression and stress. University students are considered to be particularly vulnerable to DA due to their psychological and developmental characteristics combined with the absence of direct parental supervision and a structured study environment as compared to secondary or high school, which might decrease their ability to self-regulate and cause significant distraction [11,65].

Similarly, university students could experience more psychological stress, social intimidation, or alienation during their adaptation to campus life [125]. These factors induced researchers’ interest in the possible influence of their problematic smartphone use on their academic performance. The second type of DA, social media addiction, emerged as an isolated theme during the first period, indicating that it was a significant research theme but still needed to be investigated concerning its relationship with AA during the first period. However, this relationship garnered scholarly interest during the third period, as indicated by two prominent themes: college students and social media use. In a similar bibliometric review of research addressing internet addiction in the general public, Moreno-Guerrrero et al. [126] also found that college/university students were frequently addressed in the last decade, and social media, particularly Facebook, attracted significant research interest. The study also identified a decreasing research trend into computer/video game addiction after 2010 although they continued to be investigated in fewer studies in the last couple of years.

Smartphone addiction continued to garner research interest during the second period between 2017 and 2019. However, the results indicated that research interest in the relationship between smartphone addiction and AA changed its direction from university students to high school students, and from depression and stress (psychological states) to self-control and nomophobia (mental attitudes). These results support Buctot et al.’s [127] recent remark that studies investigating the relationship between smartphone use and academic performance were conducted from a variety of perspectives, such as the associations of academic performance with the purpose of smartphone use, the time spent on smartphones, students’ self-control, behavioral intentions while engaging in mobile activities, smartphone addiction, and nomophobia. Concerning self-control, the behavioral addiction literature articulates it as one of the two main forces of addiction [3,47]. Self-control, defined as ‘the self-initiated regulation of thoughts, feelings, and actions when enduringly valued goals conflict with momentarily more gratifying goals’ [p.37] [128] is considered to be significant not only for avoiding addiction but also for maintaining grit for learning or AA [129,130,131]. As Hawi and Samaha [51] articulate, ‘it is not the smartphone per se or the mobile apps that are addictive in nature…, [but] it is a student deficiency that leads to smartphone addiction’ (p. 8). The research during the second period of analysis seemingly conveys a similar perspective. Like self-control, nomophobia, the abbreviation of ‘no-mobile-phone phobia’ [132] emerged as a recent phenomenon in peoples’ lives and manifests itself as fear of being away from one’s smartphone and their constant mental association with it, as well as unimagining a life without smartphones [31,120,133]. Research indicates that nomo31, phobia is prevalent among high school students and negatively influences their academic and social life [127,134]. These recent findings might have guided smartphone addiction research between 2018 and 2020 to address high school students and nomophobia in addition to other attitudinal problems in smartphone use.

Another finding that deserves attention is the internet addiction theme, which emerged as the sub-theme of smartphone addiction during the first period of analysis, yet lost research interest during the second period. As Duke and Montag [133] noted earlier, smartphone addiction and internet addiction often overlap because internet access makes a mobile phone ‘a smartphone’, and the internet is an integral part of smartphone usage as most of its applications require an internet connection. Some even consider smartphone addiction as a new form of internet addiction [135,136] because they replaced a multitude of devices, such as computers or tablets, with their inherent mobility and accessibility anywhere and anytime [75]. In addition, smartphones offer numerous other facilities that can be used without an internet connection, such as offline apps or games, which might stimulate addiction [45]. Indeed, in their review of the internet addiction literature, Moreno-Guerrrero et al. [126] found that smartphone/mobile phone addiction was frequently addressed in this literature, particularly after 2015. All these arguments might explain why researchers were more inclined to investigate correlations between smartphone addiction and AA.

As for the third period of analysis (2020–2022), smartphone addiction was found to have lost research interest and emerged as a declining theme. Social media addiction, network sites, and Facebook use were the most prevalent themes during this period. Social media is a means of sharing user-generated content through user-specific profiles and Web 2.0 technology and enables the development of online social networks [137,138]. It includes blogs, instant messaging platforms such as WhatsApp or Telegram, social network sites such as Facebook or Instagram, and video-sharing platforms such as YouTube [95]. With all these facilities, social media has now become an essential communication tool used by millions of people [112,139] and is considered to be not only an addiction by itself but also a significant driver of smartphone/internet addiction [22,23]. This was also supported by the results of a recent meta-analytic review, which underlined that social media use might have become an important trigger of internet addiction among young adults [140]. The study also underlined that social networking could be a coping mechanism, particularly for college students who have difficulty concentrating on their academic responsibilities. Considering these results altogether, despite the expanding research interest in the relationship between social media addiction and AA in the last couple of years, this association remains to be clarified with further investigations as existing research offers discrepant results [27,95,141]. This call was also made by some other scholars who noted that the association between social media use (or addiction) and AA was scarcely addressed in the literature despite the rising popularity of social networking among students and the concomitant increase in scholarly interest [26,30,108].

The science mapping analysis also featured some other themes that guided research on DA (i.e., smartphone addiction and social media addiction as elaborated above) and AA, depending on scholars changing perspectives on the causes and outcomes of addiction with regard to AA. For instance, during the first period, addictive behavior was a prominent theme. The research addressed student-related factors such as attention deficiency, self-control, time management skills, and video game addiction, with a particular focus on adolescents. For one thing, adolescents are primarily students and were already found vulnerable to internet addiction [142]. Conversely, video games or internet gaming distract adolescents’ attention and motivation from lessons [143] and decrease school performance [32]. This might explain increased research interest in the relationship between adolescents’ addictive behaviors and their AA.

During the subsequent period (2017–2019), interest in student-related factors continued, mainly focusing on those related to life satisfaction and self-efficacy. Life satisfaction is related to both AA and DA, and it is not easy to make causal inferences on the direction of the influence. For instance, some studies showed that higher academic performance could increase students’ life satisfaction [22]. Similarly, using digital technologies could compensate for individuals’ negative life experiences and support their satisfaction with life [144]. On the other hand, escapism and hedonic desires resulting from lower satisfaction with life could lead to DA [136].

Similarly, social media users could decrease life satisfaction by exposing them to unrealistic comparisons between themselves and others [145]. Nevertheless, studies during the second period seemingly addressed this multi-dimensional relationship between life satisfaction, DA, and AA. In addition to life satisfaction, addiction-focused research was also inclined to investigate student self-efficacy as a factor that can moderate addiction behavior and academic performance. Self-efficacy, defined as ‘people’s judgment of their capabilities to organize and execute courses of action required to attain designated types of performance’ (p. 391) [146], correlated significantly with academic performance as evidenced by earlier research, and various moderators and mediators were found to influence this relationship [147,148,149]. As a multi-dimensional concept, self-efficacy is related to motivation, emotion regulation, cognition, self-regulation, and control, while it was also found to moderate or mediate the relationship between DA and AA [58]. This might have increased research interest during the second period of analysis.

During the third period, the variety of factors investigated increased. Scholars mainly focused on family-related factors concerning adolescents’ and high school students’ intrinsic motivation and satisfaction with their academic performance, as well as the use of digital technologies in and out of school. Some scholars noted that students use digital technologies both in and out of school for different purposes, and each could have different influences on academic performance [150,151]. For instance, Luo et al. [95] found that the use of social media both inside and outside school influenced AA, and its use inside school mediated the relationship between AA and social media use outside school. Similarly, students’ family environments are considered significant in understanding their AA and their use of digital technologies [127]. Family relations or background could also mediate the relationship between DA and AA, mainly by influencing students’ motivation to learn, their academic values, and the way they use digital technologies. From this perspective, one recent study by Dou and Shek [98], for example, suggested that DA could decrease students’ intrinsic motivation to learn and have an adverse effect on their AA. On the other hand, Malik et al. [94] found that higher intrinsic motivation could mediate the positive relationship between AA and social media use.

Another prevalent theme was also found to be students’ time management skills as a significant contributor to AA through supporting self-regulated learning [152,153,154]. The overuse of digital technologies would not only replace time spent on studying and learning [141] but would also have spillover effects on time management by causing serious distraction and attention problems [22,51,108]. These recent findings call for future studies to investigate the use of digital technologies in different contexts and by different means, as well as the mediating and moderating effects of students’ different psychological and attitudinal states about learning and education.

### 4.1. Limitations

Although the current study contributed to the literature by delineating the evolving knowledge base and thematic architecture of research addressing DA and AA, some limitations should be mentioned. First, our analysis comprised WoS-indexed articles while excluding books, book chapters, or conference proceedings. Although WoS covers a broad scope of high-impact journals and the co-word analysis allowed a more extensive scope of research, our analysis might still have excluded some potentially essential studies in the field. Second, our study is different from conventional literature reviews as it combines bibliometric and science mapping analysis of the research field, with a particular focus on the intellectual development and evolution of the DA and AA research following the analysis of meta-data associated with previous studies. Therefore, it neither attempted nor provided a review of prior research findings in conventional terms. In light of our findings, though, future reviews of previous research using different methods such as meta-analysis, meta-synthesis, or systematic literature reviews could reflect the accumulated results concerning the prevalent themes in the DA–AA research field.

### 4.2. Implications for Research and Practice

DA or any of its types, such as the internet, smartphone, or social media addiction, has not been included in diagnostic tools of addiction, such as the Diagnostic and Statistical Manual (DSM) or in the International Classification of Diseases (ICD); yet, it garnered research interest and has already been accepted as a significant phenomenon in the contemporary world. Despite being a young but fast-growing and evolving field in parallel with rapid technological changes, scholars addressed many aspects of the relationship between DA and AA, as revealed by the current study. Our findings offer significant implications for the future development of this research field, mainly through reflecting on its developed or underdeveloped aspects and helping guide research interest toward the under-investigated or emerging themes.

Both DA and AA are two complex phenomena that can be moderated or altered by numerous factors, and thus investigations into their correlation naturally become even more complex, as might be implied by the discrepant or inconsistent results of existing research. Therefore, we may claim that themes from our analysis maintain their significance and warrant further investigations to enrich our understanding and strengthen the knowledge base. The themes that emerged during the last analysis period necessitate particular attention as studies addressing these themes need to be more extensive and insufficient to provide assertive results. For instance, social media or smartphones in and out of school, or for educational or other purposes, should be investigated further, perhaps comparatively, to enhance our understanding of how these technologies might hinder or facilitate students’ AA. These studies would also contribute significantly to developing educational policies or the design of technology-integrated instruction because many of these digital technologies are vital for modern education despite their addictive potential [8]. (Yang & Wu, 2012).

Similarly, recently emerging phenomena such as fear of missing information (especially about social media use) and nomophobia deserve further investigation as they become increasingly prominent among students of all ages. In addition, since previous studies are primarily cross-sectional, we cannot make casual interpretations regarding the relationships between DA and AA. Therefore, future studies in other methodologies (e.g., qualitative or SEM for mediation–moderation analysis) could provide better insights.

## Figures and Tables

**Table 1:** Inclusion/exclusion criteria.

Criteria	Included	Excluded	Rationale
*Language*	English	Other languages	International publication Authors’ ability to understand and analyze content
*Context*	All school levels/countries	Non-student populations	Broader coverage of the research field focusing on student DA–AA
*Document Type*	Journal articles	Books, book chapters, dissertations, conference proceedings	Focus on high-quality peer reviewed work
*Database*	WoS	Other databases	Comprehensive coverage of quality journals/optimum database for bibliometrics

**Table 2:** Top 10 authors most cited in the DA–AA research field.

Rank	Author	TC *	TP	h-Index
1	Hawi, Nazir S.	674	3	14
2	Samaha, Maya	674	3	9
3	Junco, Reynol	379	1	13
4	Leung, Louis	181	2	27
5	Brunborg, Geir Scott	167	1	18
6	Aune Mentzoni, Rune	167	1	17
7	Froyland, Lars Roar	167	1	4
8	Lau, Wilfred Wing-Fat	140	1	10
9	Yang, Ya-Ting	133	2	25
10	Shahzad, Basit	106	1	8

* TC: total citations; TP: total publications.

**Table 3:** Top 10 journals in terms of the number of publications on the AA–DA domain.

Rank	Journal Name	TP *	TC	JIF	JCI	Category Quartile
1	*Computers in Human Behavior*	13	1473	8.957	2.58	Q1
2	*Computers & Education*	11	521	11.182	3.75	Q1
3	*Education and Information Technologies*	5	42	3.666	1.87	Q1
4	*Journal of Behavioral Addictions*	5	316	7.772	1.39	Q1
5	*Frontiers in Psychology*	3	3	4.232	1.04	Q1
6	*Journal of Adolescence*	2	128	3.675	0.97	Q2
7	*Journal of Education and Health Promotion*	2	18	n/a	0.44	n/a
8	*Medical Teacher*	2	48	4.277	1.39	Q1
9	*Perspectives in Psychiatric Care*	2	4	2.223	0.83	Q2
10	*Social Psychology of Education*	2	11	2.614	0.80	Q2

* TP: total publications; TC: total citations; JIF: Journal Impact Factor™; JCI: Journal Citation Indicator.

**Table 4:** Top 10 articles cited in the DA–AA domain.

Rank	Article Name	Journal Name	Author(s)	Years	TC *
1	Relationships between smartphone addiction, stress, academic performance, and satisfaction with life	*Computers in Human Behavior*	Samaha, M; Hawi, NS	2016	460
2	Too much face and not enough books: The relationship between multiple indices of Facebook use and academic performance	*Computers in Human Behavior*	Junco, R	2012	379
3	Is video gaming, or video game addiction, associated with depression, academic achievement, heavy episodic drinking, or conduct problems?	*Journal of Behavioral Addictions*	Brunborg, GS; Mentzoni, RA; Froyland, LR	2014	167
4	To excel or not to excel: Strong evidence on the adverse effect of smartphone addiction on academic performance	*Computers & Education*	Hawi, NS; Samaha, M	2016	159
5	Effects of social media usage and social media multitasking on the academic performance of university students	*Computers in Human Behavior*	Lau, WWF	2017	140
6	Impact of social media usage on students’ academic performance in Saudi Arabia	*Computers in Human Behavior*	Alwagait, E; Shahzad, B; Alim, S	2015	106
7	Recognizing internet addiction: Prevalence and relationship to academic achievement in adolescents enrolled in urban and rural Greek high schools.	*Journal of Adolescence*	Stavropoulos, V; Alexandraki, K; Motti-Stefanidi, F	2013	104
8	Instant Messaging Addiction among Teenagers in China: Shyness, Alienation, and Academic Performance Decrement	*Cyberpsychology & Behavior*	Huang, HY; Leung, L	2009	91
9	Impact of Internet Literacy, Internet Addiction Symptoms, and Internet Activities on Academic Performance	*Social Science Computer Review*	Leung, L; Lee, PSN	2012	90
10	Empowering students through digital game authorship: Enhancing concentration, critical thinking, and academic achievement	*Computers & Education*	Yang, YTC; Chang, CH	2013	88

* TC: total citations.

**Table 5:** Top 10 countries with the most publications in the DA–AA domain.

Rank	Country	TP *	TC
1	China	20	526
2	USA	18	807
3	Taiwan	8	221
4	Malaysia	8	131
5	Turkey	8	94
6	England	7	252
7	Pakistan	7	67
8	Saudi Arabia	6	177
9	Spain	6	91
10	India	5	78

* TP: total publications; TC: total citations.

## Data Availability

Data used are publicly available; no identifying information was collected or included. All the data used in this research were accessed through the WoS database.
